# COX-2/PGE2 upregulation contributes to the chromosome 17p-deleted lymphoma

**DOI:** 10.1038/s41389-023-00451-9

**Published:** 2023-02-07

**Authors:** Lu Qi, Xiangyu Pan, Xuelan Chen, Pengpeng Liu, Mei Chen, Qi Zhang, Xiaohang Hang, Minghai Tang, Dan Wen, Lunzhi Dai, Chong Chen, Yu Liu, Zhengmin Xu

**Affiliations:** 1 Department of Hematology and Institute of Hematology, State Key Laboratory of Biotherapy and Cancer Center, West China Hospital, Sichuan University, Chengdu, Sichuan China; 2 State Key Laboratory of Biotherapy and Cancer Center, National Clinical Research Center for Geriatrics, West China Hospital, Sichuan University, Chengdu, Sichuan China; 3 Department of Rheumatology, North Sichuan Medical College First Affiliated Hospital, Institute of Material Medicine, North Sichuan Medical College, Nanchong, Sichuan China

**Keywords:** Cancer metabolism, Cancer genetics

## Abstract

Deletions of chromosome 17p, where *TP53* gene locates, are the most frequent chromosome alterations in human cancers and associated with poor outcomes in patients. Our previous work suggested that there were *p53*–independent mechanisms involved in chromosome 17p deletions-driven cancers. Here, we report that altered arachidonate metabolism, due to the deficiency of mouse *Alox8* on chromosome 11B3 (homologous to human *ALOX15B* on chromosome 17p), contributes to the B cell malignancy. While the metabolites produced from lipoxygenase pathway reduced, chromosome 11B3 deletions or *Alox8* loss, lead to upregulating its paralleling cyclooxygenase pathway, indicated by the increased levels of oncometabolite prostaglandin E2. Ectopic PGE2 prevented the apoptosis and differentiation of pre-B cells. Further studies revealed that *Alox8* deficiency dramatically and specifically induced *Cox-2(Ptgs2)* gene expression. Repressing *Cox-2* by its shRNAs impaired the tumorigenesis driven by *Alox8* loss. And, in turn, tumor cells with *Alox8* or 11B3 loss were sensitive to the COX-2 inhibitor celecoxib. This correlation between COX-2 upregulation and chromosome 17p deletions was consistent in human B-cell lymphomas. Hence, our studies reveal that the arachidonate metabolism abnormality with unbalanced ALOX and COX pathways underlies human cancers with 17p deletions and suggest new susceptibility for this disease.

## Introduction

Chromosome 17p is the most frequent deleted chromosome region in various human cancers, and is often associated with drug resistance and short survival [1, 2]. In lymphoid malignancies, chromosome 17p deletions (del(17p)) occur in 10-20% non-Hodgkin’s lymphoma like diffused large B-cell lymphoma (DLBCL) or mantle lymphoma, and 5-10% chronic lymphocytic leukemia [1, 3, 4]. Although del(17p) has been observed and widely used as a diagnostic marker in human cancers decades ago, its functional roles and potential underlying mechanisms are still unclear. With a conditional knockout mouse model of chromosome 11B3, the syntenic region in mice to human chromosome 17p13.1 and containing >100 coding genes, we showed that del(17p), as a whole, could promote tumorigenesis in multiple tumor models more than *p53* loss itself [2]. These results suggested that besides *TP53*, additional tumor suppressor genes (TSGs) would be in this region.

Recently, our work and others’ have shown that *PHF23*, *EIF5A*, *KCTD11*, *MAP2K4* and *HIC1* would be putative TSGs on chromosome 17p [5–8]. To better understand the tumor suppression mechanisms of chromosome 17p, we performed an in vivo tumorigenesis screening with an shRNA library against the 99 coding genes on chromosome 11B3 in mice. Multiple potential TSGs were scored in this screening, including the plant homeodomain finger protein 23 (*PHF23)* and arachidonate 15-lipoxygenase type B (*ALOX15B*) [2, 8]. PHF23 is a reader for histone 3 lysine 4 tri-methylation and negatively regulates the deacetylase activity of HDAC through a new epigenetic regulatory complex, the PSH complex. Thus, the PSH complex coordinates two active histone markers, H3K4me3 and H3K27ac, for gene activation. Loss of *PHF23* represses the expressions of differentiation-related genes and other downstream TSGs and thus promotes tumorigenesis [8]. Besides this PHF23-regulated epigenetic mechanism, metabolic alterations have been observed in cancers with del(17p). *ALOX15B* deficiency leads to accumulation of its substrate, arachidonic acid (AA), and accelerates *Myc*-driven lymphoma. Interestingly, these 17p TSGs collaborate with p53 to inhibit tumorigenesis, which might contribute to the super tumor suppression capacity of chromosome 17p as a whole [9]. However, the metabolic mechanism in cancer cells with del(17p) or *ALOX15B* loss has not been well understood. ALOX15B is a member of iron-containing polyunsaturated fatty acid metabolism enzymes, which oxygenate AA and generate a series of products, including hydroperoxy eicosatetraenoic acids (HpETEs), hydroxyeicosatetraenoic acid (HETEs) and leukotrienes. AA is a polyunsaturated fatty acid and abundant in many types of cells. AA can be metabolized through three distinct pathways, the cyclooxygenase pathway, the lipoxygenase pathway and the cytochrome P450 pathway, into numerous biologically active metabolites. In humans, there are six lipoxygenases and interestingly, five of them, *ALOX15B*, *ALOX12B*, *ALOXE3*, *ALOX12* and *ALOX15*, reside in chromosome 17p. AA metabolism has been implicated in multiple biological processes and human diseases, such as inflammation, myocardial infarction, cholesterol metabolism, and cancer [10–12]. *ALOX15B* and *ALOX12* in lymphoma have been reported to have a tumor-suppressive role in promoting murine lymphomas, while *ALOX5*, the only one not on chromosome 17p, seems to be required at least for some types of leukemia [2, 11, 13]. In contrast, cyclooxygenases, especially COX-2, and their products, such as prostaglandin E2 (PGE2), seem to have oncogenic roles in various types of cancers [14, 15]. Given these paradoxical functions of AA metabolism, it would be interesting to investigate how AA metabolism is disturbed in del(17p) and *ALOX15B* deficient tumors and their potential functions in tumorigenesis and treatment.

In this study, we profiled the metabolites derived from AA in del(17p) and *ALOX15B* deficient lymphoma cells by liquid chromatography-mass spectrometry (LC-MS). We analyzed the in vitro and in vivo functions of the cyclooxygenase pathway in these tumors with genetic and pharmaceutic assays. We found that upregulating the cyclooxygenase pathway contributed to del(17p) cancers’ biology and implied new vulnerability for this disease.

## Results

### Prostaglandin E2 is specifically enriched in del(17p) and *Alox8*-deficient cells

**Fig. 1:**
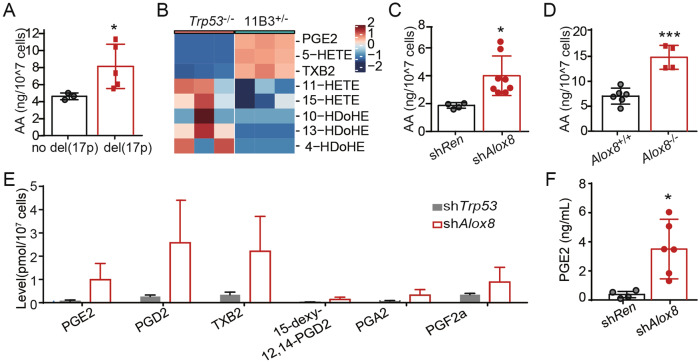
Prostaglandin E2 was upregulated in mouse *Alox8*- or chromosome 11B3 deleted lymphomas. **A** AA levels in fresh patients’ white blood cells enriched with chromosome 17p-deleted CLL cells, compared to those without 17p deletion, measured by liquid chromatography-mass spectrometry (LC-MS). *n* = 3 for no del(17p) group and *n* = 5 for del(17p) group. Error bar represents “Mean with SEM”, *p < 0.05 (unpaired two-tailed *t-*test). **B** Profiling AA metabolites in mouse primary lymphoma cells with chromosome 11B3 deletion (11B3^+/-^), compared to *Trp53*^*-/-*^ lymphoma cells, by LC-MS (*n* = 3). **C** AA levels in sh*Alox8* vs sh*Ren* Ba/F3 cells, measured by LC-MS and ELISA, respectively. *n* = 4 for sh*Ren* group and *n* = 8 for sh*Alox8* group. Error bar represents “Mean with SEM”, *p < 0.05 (unpaired two-tailed *t-*test). **D** AA levels in *Alox8*^*+/+*^ vs *Alox8*^-/-^ Ba/F3 cells measured by LC-MS and ELISA, respectively. *n* = 6 for Alox8^+/+^ group and *n* = 4 for *Alox8*^-/-^ group. Error bar represents “Mean with SEM”, ***p < 0.001 (unpaired two-tailed *t-*test). **E** Profiling AA metabolites in primary sh*Alox8* lymphoma cells, compared to sh*Trp53* lymphoma cells, by LC-MS (*n* = 3). **F** PGE2 levels in *Myc*; sh*Trp53***-**sh*Ren* (sh*Ren*) or *Myc*; sh*Trp53*-sh*Alox8* (sh*Alox8*) lymphoma cells, measured by ELISA. *n* = 4 for sh*Ren* group and *n* = 6 for sh*Alox8* group. Error bar represents “Mean with SEM”, *p < 0.05 (unpaired two-tailed *t-*test).

Our previous work has shown that arachidonic acid (AA) was accumulated in murine B-cell lymphomas with *Alox8*-knockdown or chromosome 11B3 deletion, which is syntenic to human chromosome 17p13 [2]. Here, we collected peripheral white blood cells from B-cell chronic lymphocytic leukemia (B-CLL) patients at West China Hospital, and measured AA amount through liquid chromatography-mass spectrometry (LC-MS). We found that cellular AA levels were significantly higher in del(17p) CLL (*n* = 3) than no del(17p) ones (*n* = 5) (Fig. 1A). Given that AA can be oxygenated into diverse bioactive lipid metabolites, we wondered how these elevated AA levels would alter the AA-derived metabolites in del(17p) tumors. Therefore, we profiled the AA metabolites, produced through either lipoxygenase or cyclooxygenase pathway, in primary mouse B-cell lymphomas with or without 11B3 loss by LC-MS. As Fig. 1B shown, two AA derivatives oxygenated by the cyclooxygenase pathway, prostaglandin E2 (PGE2) and thromboxane B2 (TXB2), were increased in 11B3-deleted lymphoma cells, while multiple metabolites produced from arachidonate lipoxygenases, including hydroxyeicosatetraenoates (HETEs) were reduced in 11B3-deleted cells compared to *Trp53* loss only lymphoma cells. Interestingly, the levels of several other ALOX products, such as several hydroxy docosahexaenoic acids (HDoHEs) derived from docosahexaenoic acid (DHA) were decreased. Altogether, the AA metabolism is largely disrupted in del(17p) tumor cells, with a downregulated lipoxygenase pathway and upregulated cyclooxygenase pathway.

Given that there are five ALOX genes on chromosome 17p, next we tested whether *ALOX15B* loss would be able to mimic del(17p) and directly led to the AA metabolism dysregulation. In a previous study, we showed that AA was increased in mouse NIH3T3 fibroblast cells with Alox8 knockdown, homologous to human *ALOX15B* [2]. Here, we confirmed this observation in Ba/F3 cells, a murine pro-B cell line that is more relative to B cell lymphoma. Ba/F3 cells infected with sh*Alox8* had significantly higher levels of AA than that in cells with sh*Ren*, measured by LC-MS (Fig. 1C). Similar results of higher AA levels were observed in the corresponding medium of Ba/F3 cells with sh*Alox8* (Supplementary Fig. 1A). Further, we generated Ba/F3 cells with *Alox8* loss-of-function mutations by CRISPR/Cas9 technology, verified by T7 endonuclease I assay and Sanger sequencing (Supplementary Fig. 1C and D). The cellular and medium AA levels were significantly higher in *Alox8*-mutated cells (*Alox8*^*-/-*^) than in control cells (Fig. 1D and Supplementary Fig. 1B), consistently with what we observed in sh*Alox8* cells. Then we profiled the AA metabolites by LC-MS in primary B-cell lymphoma cells with or without sh*Alox8*. In consistent with the finding in del(11B3) lymphoma cells, multiple AA downstream metabolites produced from cyclooxygenase pathway, such as PGE2, PGD2 (Prostaglandin D2) and TXB2, were upregulated in *Alox8*-deficient lymphoma cells (sh*Alox8*;*Myc*), compared with control lymphoma cells (sh*Trp53*;*Myc*) (Fig. 1E). The accumulation of PGE2 in tumor cells with *Alox8* loss was further confirmed by enzyme-linked immunosorbent assay (ELISA) (Fig. 1F). These data suggested that *Alox8* loss contributed to the enrichment of AA and it derives like PGE2 via cyclooxygenase pathway in tumor cells with del(17p).

### RNA-seq analyses reveal a transcriptomic similarity between *Alox8*-deficient cells and those treated with PGE2

**Fig. 2:**
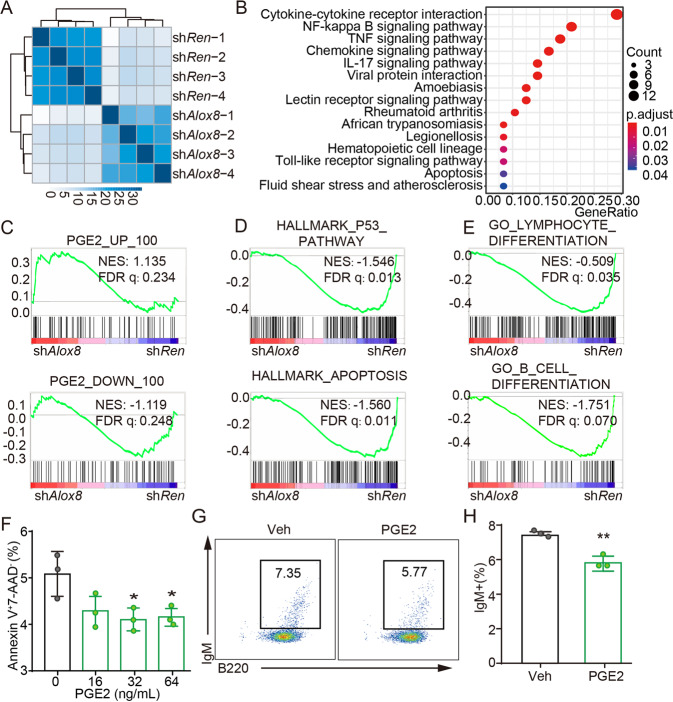
Transcriptome analysis revealed that inhibition of *Alox8* has similar gene expression as PGE2-treated cells. **A** Unsupervised clustering of RNA-seq data of sh*Alox8* or sh*Ren* pre-B cells. *n* = 4, **B** KEGG enrichment analysis of genes downregulated in sh*Alox8* cells comparing to sh*Ren* cells. **C**–**E** Gene set enrichment analysis of genes belonging to the top 100 differentially expressed genes with PGE2 treatment (**C**), HALLMARK_P53_PATHWAY and HALLMARK_APOPTOSIS gene sets (**D**) or GO_LYMPHOCYTE_DIFFERENTIATION and GO_B_CELL_DIFFERENTIATION gene sets (**E**) in sh*Alox8* cells comparing to sh*Ren* cells. NES, normalized enrichment score; FDR, false discovery rate. **F** The percentage of Annexin V + 7-AAD- apoptotic cells of pre-B cells treated with different concentrations of PGE2, measured by flow cytometry. *n* = 3. *p < 0.05 (unpaired two-tailed *t*-test). **G**, **H** The percentage of IgM+ in B220 + B cells as measured by flow cytometry analysis. *n* = 3. Error bar represents “Mean with SEM”, **p < 0.01(unpaired two-tailed *t*-test).

To decipher the molecular consequences of *Alox8* loss, we analyzed the transcriptomes of pre-B cells with *Alox8* or control *Renilla* shRNAs by RNA sequencing (RNA-seq). Both the unsupervised hierarchical clustering and principal component analysis (PCA) plot showed that the RNA-seq results of pre-B cells with sh*Alox8* were grouped together, distinguished from sh*Ren* cells (Fig. 2A and Supplementary Fig. S2A). Gene ontology (GO) terms and Kyoto Encyclopedia of Genes and Genomes (KEGG) pathways enrichment analysis show that 154 GO terms and 26 KEGG pathways were significantly downregulated in sh*Alox8* pre-B cells, compared to control sh*Ren* cells. Among them were immune response-related pathways such as cytokine-cytokine receptor interaction, chemokine signaling pathway, cellular response to interleukine-1, response to lipopolysaccharide, and the apoptosis pathway significantly downregulated in sh*Alox8* cells (Fig. 2B and Supplementary Fig. S2B). Gene set enrichment analysis (GSEA) results revealed that the upregulated genes by PGE2 treatment were positively enriched in sh*Alox8* pre-B cells, compared to sh*Ren* cells (NES = 1.135). In contrast, the downregulated genes by PGE2 treatment were negatively enriched (NES = −1.119) (Fig. 2C) [16]. The transcriptomic similarity further confirmed the altered cyclooxygenase pathway by *Alox8* loss.

### PGE2 increase prevents cellular apoptosis and B-cell differentiation

Gene set enrichment analysis (GSEA) results also showed that HALLMARK_P53 PATHWAY (NES = −1.546, FDR *q* = 0.013) and HALLMARK_APOPTOSIS pathway gene sets (NES = −1.560, FDR *q* = 0.011) were significantly negatively enriched in the transcriptome of sh*Alox8* cells compared to that of sh*Ren* cells (Fig. 2D). Multiple lymphocyte differentiation and B cell differentiation gene signatures were negatively enriched in *Alox8*-deficient pre-B cells (Fig. 2E). The enrichment of these pathways might underlie the anti-apoptosis and differentiation block effects of *Alox8* deficiency.

To test whether upregulated PGE2 by *Alox8* loss contributed to apoptosis and B-cell differentiation, we treated pre-B cells, the assumable cell-of-origin for *Myc*-driven B lymphoma in our model, with PGE2. The results showed that ectopic PGE2 prevented the apoptosis of pre-B cells, measured by Annexin V and 7-AAD staining, in a dosage-dependent manner (Fig. 2F). Further, we found that the differentiation of pre-B cells, indicated by IgM expression, was significantly repressed by PGE2 treatment (Fig. 2G, H). The survival advantage and differentiation block suggested that PGE2 might promote the transformation of B progenitor cells.

### *Cox-2 (Ptgs2)* but not *Cox-1 (Ptgs1)* is induced by *Alox8* deficiency

**Fig. 3:**
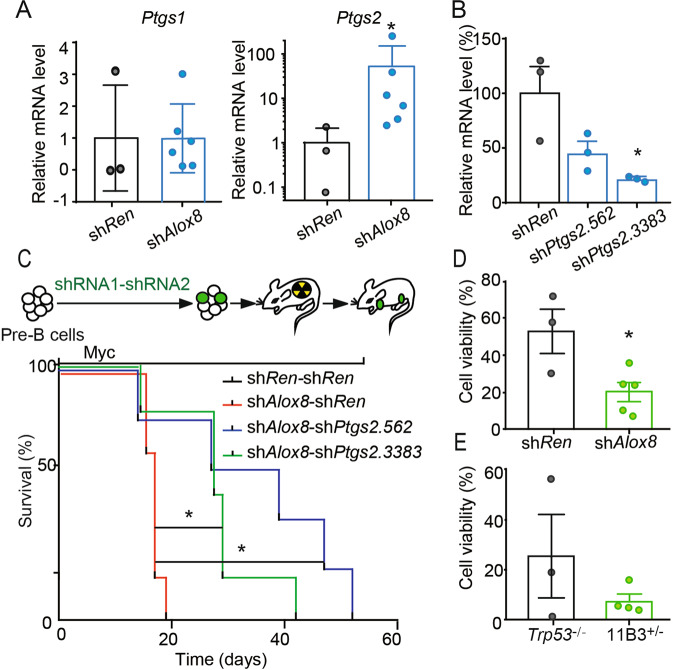
*Ptgs2* was upregulated in *Alox8*-deficient lymphomas, which contributed to lymphomagenesis. **A** Relative mRNA levels of *Ptgs1(Cox-1)* and *Ptgs2(Cox-2)* in sh*Ren* or sh*Alox8* lymphoma cells, measured by RT-qPCR. *n* = 3 for sh*Ren* group and *n* = 6 for sh*Alox8* group. *p < 0.05 (un*p*aired two-tailed *t*-test). **B** Knockdown efficiency of *Ptgs2* by sh*Ptgs2*.562 and sh*Ptgs2*.3383 in sh*Trp53* lymphoma cells, measured by RT-qPCR. *n* = 3. Error bar represents “Mean with SEM”, *p < 0.05 (unpaired two-tailed *t*-test). **C** Kaplan–Meier tumor-free survival of recipient mice transplanted with tandem shRNAs for both *Alox8* and *Ptgs2*. *n* = 6. *p < 0.05 (log-rank test). **D** Relative cell counts of *Myc*; sh*Trp53*-sh*Ren* (sh*Ren*) or *Myc*; sh*Trp53*-sh*Alox8* (sh*Alox8*) lymphoma cells treated with 50 μM celecoxib or vehicle for 48 h. *n* = 3 for sh*Ren* group and *n* = 5 for sh*Alox8* group. Error bar represents “Mean with SEM”, *p < 0.05 (unpaired two-tailed *t-*test). **E** Relative cell counts of *Trp53*^*-/-*^ or 11B3^+/-^ lymphoma cells treated with 50μM celecoxib or vehicle for 48 h. *n* = 3 for sh*Ren* group and *n* = 4 for sh*Alox8* group.

Cyclooxygenase is the key enzyme to catalyze AA into prostaglandins, including PGE2. There are two cyclooxygenases COX-1 and COX-2, encoded by *PTGS1* and *PTGS2*, respectively. It is known that while *PTGS1* is constitutively-expressed, *PTGS2* gene transcription could be rapidly induced in response to stimuli [17]. Consistent with the increased levels of the cyclooxygenase pathway metabolites, we found that *Ptgs2* but not *Ptgs1* gene expression was dramatically induced up to 250-fold in *Alox8*-deficient lymphoma cells, compared to that in control sh*Ren* lymphoma cells (Fig. 3A). The synergistically increased expressions of *PTGS2*, *PTGES* and its metabolite PGE2 indicated a shift of the AA metabolism from the lipoxygenase pathway to the cyclooxygenase pathway.

### Upregulation of cyclooxygenase pathway is required for *Alox8* loss-driven lymphomagenesis

Because of the disrupted AA metabolism balance between the lipoxygenase pathway and the cyclooxygenase pathway in *Alox8*-deficient tumors and the known oncogenic functions of PGE2 in various cancers, we hypothesized that the enhanced cyclooxygenase pathway might mediate the tumor-promoting function of *Alox8* loss. Two independent shRNA against *Ptgs2* were cloned and validated by qRT-PCR (Fig. 3B). *Ptgs2* shRNAs were cloned into a retrovirus-based vector carrying *Myc* cDNA and tandem shRNAs (sh*Alox8.2865*-sh*Ptgs2.562* or sh*Alox8.2865*-sh*Ptgs2.3383*), with which *Alox8* and *Ptgs2* would be simultaneously repressed in the same cell with *Myc* overexpression. These tandem shRNA constructs were introduced into B progenitor pre-B cells, followed by transplantation into sub-lethally irradiated recipient C57BL/6 mice. The recipient mice were monitored daily by palpation, and the tumor-free survivals were recorded. Kaplan–Meier curve showed that recipient mice transplanted with sh*Alox8.2865*-sh*Ren* transduced pre-B cells developed lymphomas with a median latency of 17 days, similar to those with sh*Alox8.2865* only [2]. However, compared to control sh*Alox8*-sh*Ren* recipient mice, mice carrying sh*Alox8*-sh*Ptgs2* pre-B cells developed lymphomas with significantly longer tumor latencies (Fig. 3C; Median onset 27 and 39 days, respectively). Intriguingly, although the resulting lymphoma cells containing sh*Alox8*-sh*Ptgs2* as enriched GFP + cells in enlarged lymph nodes, these cells have upregulated *Ptgs2* gene expression to a similar level as that in control sh*Alox8*-sh*Ren* lymphoma cells (data not shown) and displayed similar histological features (Supplementary Fig. 3A, B), suggesting that sh*Ptgs2* suppressive effect on *Ptgs2* mRNA level had been bypassed or compensated during lymphomagenesis. Altogether, these results strongly indicated that the cyclooxygenase pathway upregulation, following *Alox8* loss, was required for lymphomagenesis driven by *Alox8* deficiency.

The AA metabolism alteration with the lipoxygenase-cyclooxygenase pathway unbalances implied that a gain of vulnerability for cancers with del(17p) or *ALOX15B* loss. As a proof-of-concept, we treated lymphoma cells with sh*Ren* or sh*Alox8* with celecoxib, a commonly used anti-inflammation drug inhibiting COX-2. The results showed that sh*Alox8* tumor cells were significantly more sensitive to COX-2 repression than the control cells (Fig. 3D). Further, we tested the inhibitory effect of celecoxib on mouse del(11B3) lymphoma cells. Tumor cells with del(11B3) also displayed a better response to celecoxib treatment than those with only p53 loss (Fig. 3E). The susceptibility of these *Alox8* deficient tumor cells to celecoxib suggested that targeting the unbalanced AA metabolism might be a new treatment for cancers with del(17p) in patients.

### The unbalanced lipoxygenase pathway and cyclooxygenase pathway in lymphoma patients with del(17p) suggest new susceptibility

**Fig. 4:**
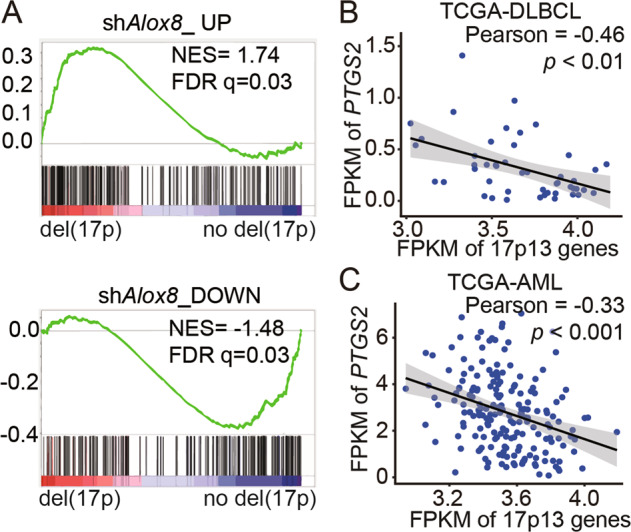
Upregulation of cyclooxygenase pathway in lymphoma patients with del(17p) suggest new susceptibility. **A** GSEA plots showing that genes were chosen from top 200 upregulated (sh*Alox8*_UP) or downregulated (sh*Alox8*_DOWN) genes in sh*Alox8* cells comparing to sh*Ren* cells significantly positively or negatively enriched in human chromosome 17p-deleted DLBCL cells. **B**, **C** Correlations between expression levels of *PTGS2* and averages of chromosome 17p genes in TCGA-DLBCL or TCGA-AML. Data analyzed from TCGA-DLBCL (*n* = 48) or TCGA-AML (*n* = 187).

Given that there are multiple tumor suppressor genes like *p53* and *PHF23* on chromosome 17p, we wondered how much *ALOX15B* repression and the resulted aberrant AA metabolism would contribute to del(17p) cancer. GSEA results showed that the genes upregulated in sh*Alox8* cells were significantly positively enriched in human chromosome 17p-deleted DLBCL cells (NES = 1.74, FDR *q* = 0.03). And in contrast, the genes downregulated in sh*Alox8* cells were significantly negatively enriched in del(17p) DLBCL (NES = −1.48, FDR *q* = 0.03) (Fig. 4A). These results suggest that *Alox8* loss partially mimicked the molecular characteristics of del(17p). The cyclooxygenase pathway was upregulated in both *Alox8* deficient and 11B3-deleted mouse lymphoma cells, indicated by increased levels of *Ptgs2* expression and its product PGE2 (Figs. 1E and 1F). Here, we further tested the expressions of *PTGS2* in human del(17p) tumors. Since the expression levels of genes on chromosome 17p are reduced in 17p-deleted tumors, we used the corrected expression levels of chromosome 17p13 genes to indicate 17p deletions. By analyzing the transcriptomes of 48 DLBCL samples in the TCGA cohort, we found that the expression levels of *PTGS2* were significantly negatively correlated with those of chromosome 17p13 genes (Pearson = −0.46, *p* < 0.01) (Fig. 4B). A similar correlation was also observed in the TCGA-AML cohort (Pearson = −0.33, *p* < 0.001) (Fig. 4C). The negative correlation between 17p genes and *PTGS2* was consistent with our hypothesis that the cyclooxygenase pathway of AA metabolism was upregulated in del(17p) cancers.

**Fig. 5:**
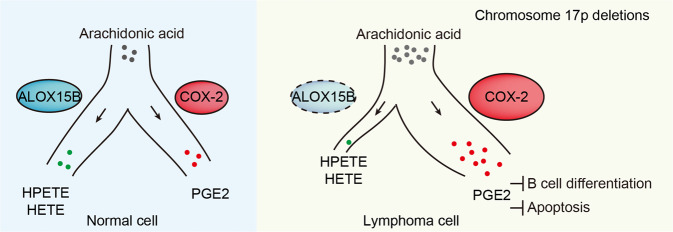
Unbalanced arachidonate lipoxygenase and cyclooxygenase pathway in 17p-deleted lymphomas. Schematic representation showing that arachidonate lipoxygenase and cyclooxygenase pathway in normal cell (left) or lymphoma cells with chromosome 17p deletions (right).

Taken together, our studies revealed the AA metabolism alterations in del(17p) cancers. The deficiency of the co-deleted 17p TSG *ALOX15B* led to a repressed lipoxygenase pathway and an enhanced cyclooxygenase pathway for AA metabolism (Fig. 5). Moreover, this metabolism abnormality implied a new susceptibility to this recalcitrant malignancy.

## Discussion

Metabolism reprogramming has been shown to be a hallmark of cancer as early as a century ago when Warburg’s effect was first described [18, 19]. Besides numerous studies on glucose metabolism, there are emerging evidences showing that altered lipid metabolism has significant effects on tumorigenesis not only through providing essential cellular components but also as signaling molecules [20–22]. AA, a polyunsaturated fatty acid with a 20-carbon chain, can be converted into multiple bioactive lipids through three distinct metabolism pathway, the lipoxygenase pathway, the cyclooxygenases pathway and the cytochrome P450 pathway. Some of these AA-derived products have been shown to be involved in inflammation, cancer and other human diseases [23]. In this study, we have illustrated that cyclooxygenases/prostaglandins, upregulated by lipoxygenase *ALOX15B* loss, contributed to the tumor formation and maintenance of *Alox8*-deficient lymphoma. COX-2 is known to be generally absent in most normal cells but is highly enriched in many malignant cells like colorectal, lung, breast and prostate cancers; and PGE2, the major bioactive metabolite of COX genes, can promote the proliferation of cancer cells, enhance their stemness and protect them from apoptosis [24]. Furthermore, the high expression of *PTGS2*, encoding COX-2, is also correlated with poor prognosis in lymphomas [25]. In contrast, *ALOX15B* is recognized to have an anti-carcinogenic effect as its downregulated expression in various cancers and its loss driving lymphoma development [2, 26–28]. Our work revealed that these two enzymes can crosstalk. Loss of *Alox8* specifically induced *Ptgs2* expression, inhibiting which would prevent *Alox8*-deficient B progenitor cells transformed into lymphoma, and suppress *Alox8*-deficient lymphoma cell growth. *PTGS2* is suggested to be induced in response to various cellular and environmental stresses. There are some signal pathways like NF-κB and PI3K/AKT pathway reported to regulate *PTGS2* gene transcription [29, 30]. We found that *Alox8* loss activated the PI3K-mTOR pathway (data not shown). But, whether the upregulated PI3K-mTOR pathway is downregulated *Alox8* cell induces *PTGS2* gene expression needs future study.

Chromosome 17p deletions are one of the most frequently deleted chromosome regions that happened in almost all known cancers. Our previous work and other laboratory studies have demonstrated that chromosome 17p deletions have an impact on cancer biology beyond *TP53* loss. Multiple tumor suppressor genes like *PHF23, EIF5A*, *KCTD11*, *MAP2K4* and *ALOX15B*, have been reported to promote tumorigenesis when lost, indicating that gene expression regulation, signal transduction, arachidonate lipoxygenase and other cellular pathways are altered with the loss of chromosome 17p [2, 5, 6, 8]. Here, our results indicate that the arachidonate cyclooxygenase pathway also plays a role in 17p-deleted tumor biology. We showed that *ALOX15B* deficiency with chromosome 17p deletions was demonstrated to promote lymphomagenesis and maintain corresponding lymphoma cell growth through upregulating *COX-2* level, as discussed before. Moreover, high expression of *COX-1* and especially *COX-2* are correlated with chromosome 17p loss. Loss of mouse chromosome 11B3, homologous to human chromosome 17p13, has a high level of the product of the cyclooxygenase pathway, PGE2. More importantly, 11B3-deleted lymphoma cells were more sensitive to COX-2 inhibitor celecoxib. These results support that arachidonate cyclooxygenase/prostaglandins contribute to the chromosome 17p-deleted cancer biology. How these various dysregulated pathways in chromosome 17p deletions cooperatively work during the progression of malignancy and response to commercial therapies are under study.

Besides *ALOX15B*, there are four other arachidonate lipoxygenases locating on chromosome 17p, including *ALOX12*, *ALOX12B*, *ALOX15* and *ALOXE3*. Among them, *Alox12* was reported to be required for the tumor suppression functions of p53 in an *Eµ-Myc* mouse lymphoma model [13]. Consistently, we found knockdown *Alox12* could drive Myc-overexpressed pre-B cells transformed into lymphoma in recipient mice (data not shown). These tumor-suppressive ALOX genes are clustered in chromosome 17p suggested that their loss in 17p-deleted cancers may have a cooperative impact on tumor development. Conversely, the only ALOX family gene outside of chromosome 17p, *ALOX5* on chromosome 10, did not have the same tumor-suppressing role in our mouse lymphoma model. Instead, other reports show that ALOX5 may have opposite roles in a different context that it promoted BCR-ABL driven B-cell acute lymphoblastic leukemia while inhibited MLL-fusion driven acute myeloid leukemia [11, 31]. Interestingly, ALOX5 product 5-HETE was significantly increased in mouse chromosome 11B3-deleted lymphoma cells compared to that in *Trp53*^-/-^ control. Inhibition of ALOX5 has a synthetic effect with COX-2 inhibitor on suppressing cancer cell growth. Therefore, it may be a promising therapeutic strategy to treat 17p-deleted lymphomas with both ALOX5 and COX-2 inhibitors.

## Materials and methods

### Mice

All mice experiments were approved by the Institutional Animal Care and Use Committees of Sichuan University. Mouse lymphomagenesis experiments were performed as previously described [2]. Briefly, B220 + B progenitor cells isolated from bone marrow were transduced with retroviruses followed by tail-vein injection into sublethally irradiated C57BL/6 mice. Lymphomas were monitored weekly with disease state being defined by palpable enlarged solid lymph nodes. Tumor measurements were performed blindly. All recipient mice were divided into each group randomly before transplantation. Tumor measurements were performed blindly.

### Human samples

Primary peripheral blood cells from patients with CLL were collected at the outpatient department of West China Hospital during 2017–2018. The status of chromosome 17 was confirmed by fluorescence in situ hybridization (FISH). A total of 10 patients were recruited, including 6 with 17p deletion, and 4 with intact 17p. Peripheral blood samples were transported at 4 °C and lysed using ACK lysing buffer within 24 h. After lysing, 2 × 10^6^ white blood cells were prepared for intracellular lipids extraction. The Ethics Committee approved the study of West China Hospital of Sichuan University [HX-2019-(114)].

### Cell line culture

Ba/F3 (#HB-283), 293 T (#CRL-1573) were from ATCC. Ba/F3 cells were maintained in RPMI 1640 medium supplemented with 10% v/v FBS and 2 ng/mL IL-3(cat. No.:CP39; Novoprotein). 293 T cells were maintained in DMEM medium supplemented with 10% v/v FBS. All cell lines were routinely tested for *Mycoplasma* by PCR. Experiments were performed within 4 weeks after fresh viable cells were thawed.

### Retroviral construction

shRNAs were cloned into MSCV-mirE-SV40-Myc-IRES-GFP retroviral construct. Retrovirus packaging and infection of cells were done as previously reported [2].

### ELISAs

Prostaglandin E2 (PGE2) was measured using an ELISA kit (cayman, CAS 363-24-6) according to the manufacturer’s instructions. Arachidonic acid (AA) was measured using an ELISA kit (Elabscience, E-EL-0051c) according to the manufacturer’s instructions.

### LC-MS

Cells were extracted with chloroform/methanol and washed with 0.9% saline. The lipid-containing chloroform phase was obtained. Standard curves were constructed by least-squares linear regression analysis using the peak area ratio of a given eicosanoid over its reference IS against the calibrator’s nominal concentration. UPLC-MS/MS analyses were conducted on an Agilent UPLC-MS/MS system consisting of 1290 UPLC-system coupled with an Agilent 6470 triple-quadrupole mass spectrometer (Agilent Technologies, USA). Peak determination and peak area integration was performed with MassHunter Workstation software (Agilent, Version B.08.00), while auto-integration was manually inspected and corrected if necessary. Appropriate internal standards corrected the obtained peak areas of targets (IS) and calculated response ratios were used throughout the analysis. All experiments were repeated more than twice.

### RNA-sequencing analysis

RNA-seq were sequenced by BGISEQ500 sequencing machine with 50-bp single-end reads. The RNA-seq reads were aligned to the reference genome (GRCm38) by STAR_2.6.0a. Transcript abundance was normalized and measured in Transcripts Per Kilobase Million (TPM). Differential gene expressions were analyzed by DESeq2. Genes with absolute fold changes >1 and FDR ≤ 0.05 were counted as differentially expressed genes. Samples distance were calculated by PCA and the Euclidean distance. GSEA was applied to show statistically significant similarities and differences between two given clusters by identifying a priori-defined gene set. Our model was ordered to obtain a rank-ordered gene list and the top 100 high and low expressed genes in the gene set GSE1195214 and GSE209445 as grp files. Followed by running GSEA, TCGA DLBCL patients split into 17 P deletion and no 17 P deletion, 17 P deletion VS no 17 P deletion Fold change to obtain a rank-ordered gene list, and the top 200 high and low expressed genes in sh*Alox8*. Followed by running GSEA.

All the transcriptome data in the TCGA database were transformed by log2(X + 1). Correlations between PTGS2 expression levels and the mean expression levels of 97 genes in 17p detected in TCGA-LAML and TCGA-DLBC were calculated by Pearson’s correlation coefficient and statistic powers were quantified followed by a two-sided hypothesis test and 0.95 confidence level.

### Quantitative PCR

RNA was isolated with Trizol, cDNA was synthesized with QuantStudio™ 3 Real-Time PCR System (Thermo Fisher Scientific). All experiments were repeated more than twice. A list of all primers used for PCR analysis is given in Supplementary Table 1.

### In vitro drug response assays

Lymphoma cell lines generated from sh*Trp53*-sh*Ren*, sh*Trp53*-sh*Alox8.1252*, sh*Trp53*-sh*Alox8.2865* tumor-bearing mice were cultured in BCM medium (45% DMEM, 45% IMDM, 10% FBS, 2 mM glutamine, 50 μM β- mercaptoethanol, 1× penicillin/streptomycin) in 96-well plates. Cells were treated with the indicated concentrations of celecoxib (Selleck; S1261) for 2 days. The celecoxib dissolved in DMSO. The number of living cells was determined by BD Accuri C6. All experiments were repeated more than twice.

### Statistical analysis

Statistical analysis was performed by GraphPad Prism9 (RRID: SCR_002798). Kaplan–Meier tumor-free survival were analyzed by log-rank test. Other statistical significance was examined by unpaired two-tailed *t*-test.

## Supplementary information

Supplemental figure 1

Supplemental figure 2

Supplemental figure 3

Supplementary figure legends

Supplemental tables

## Data Availability

All data generated or analyzed during this study are included.
