# Supplementary figures and images for: COX-2/PGE2 upregulation contributes to the chromosome 17p-deleted lymphoma
Source: Oncogenesis. 2023 Feb 7;12(1):5. doi: 10.1038/s41389-023-00451-9 (PMC9905509; doi:10.1038/s41389-023-00451-9)

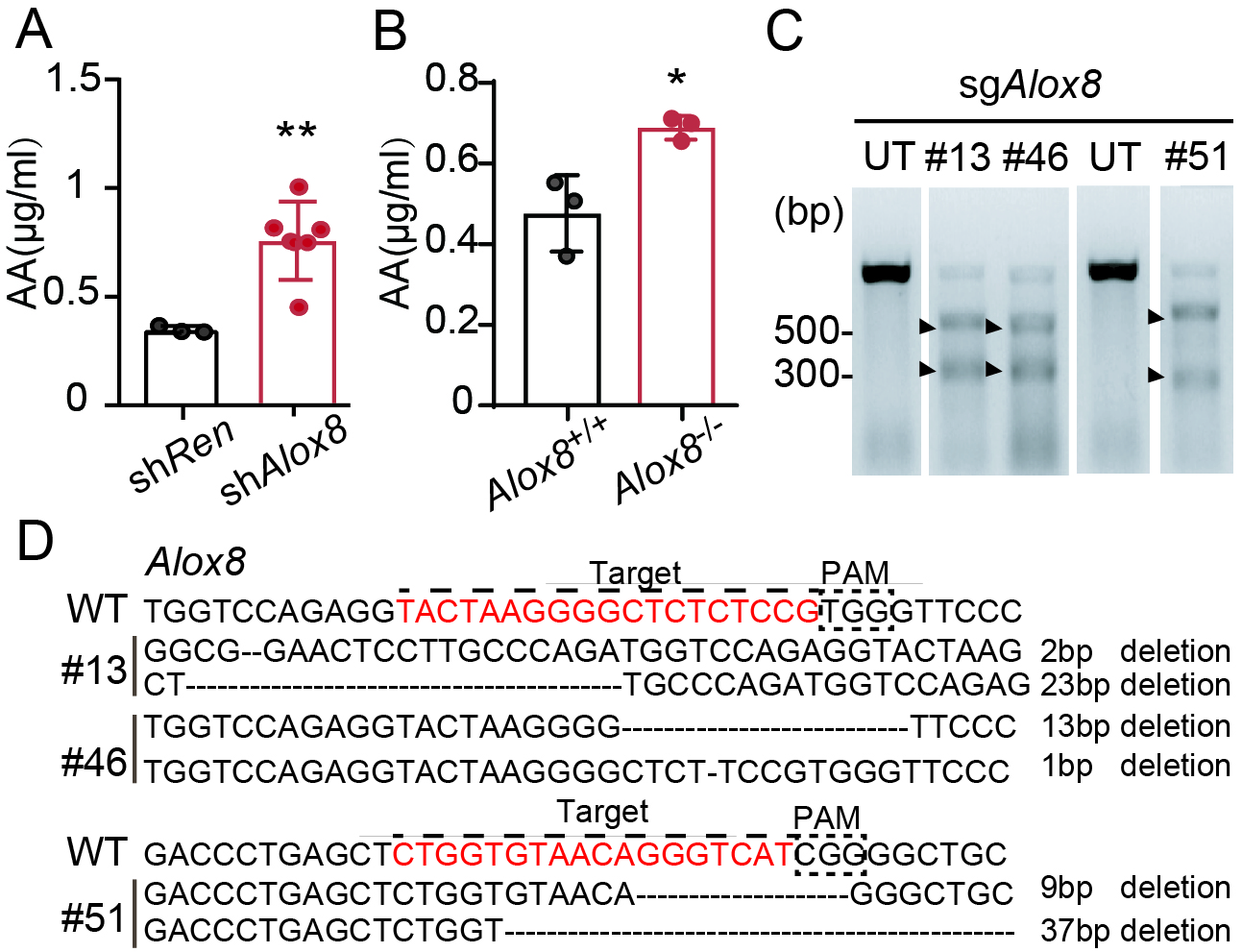

Supplement: Supplementary file 1 — Supplemental figure 1 [file 41389_2023_451_MOESM1_ESM.jpg]

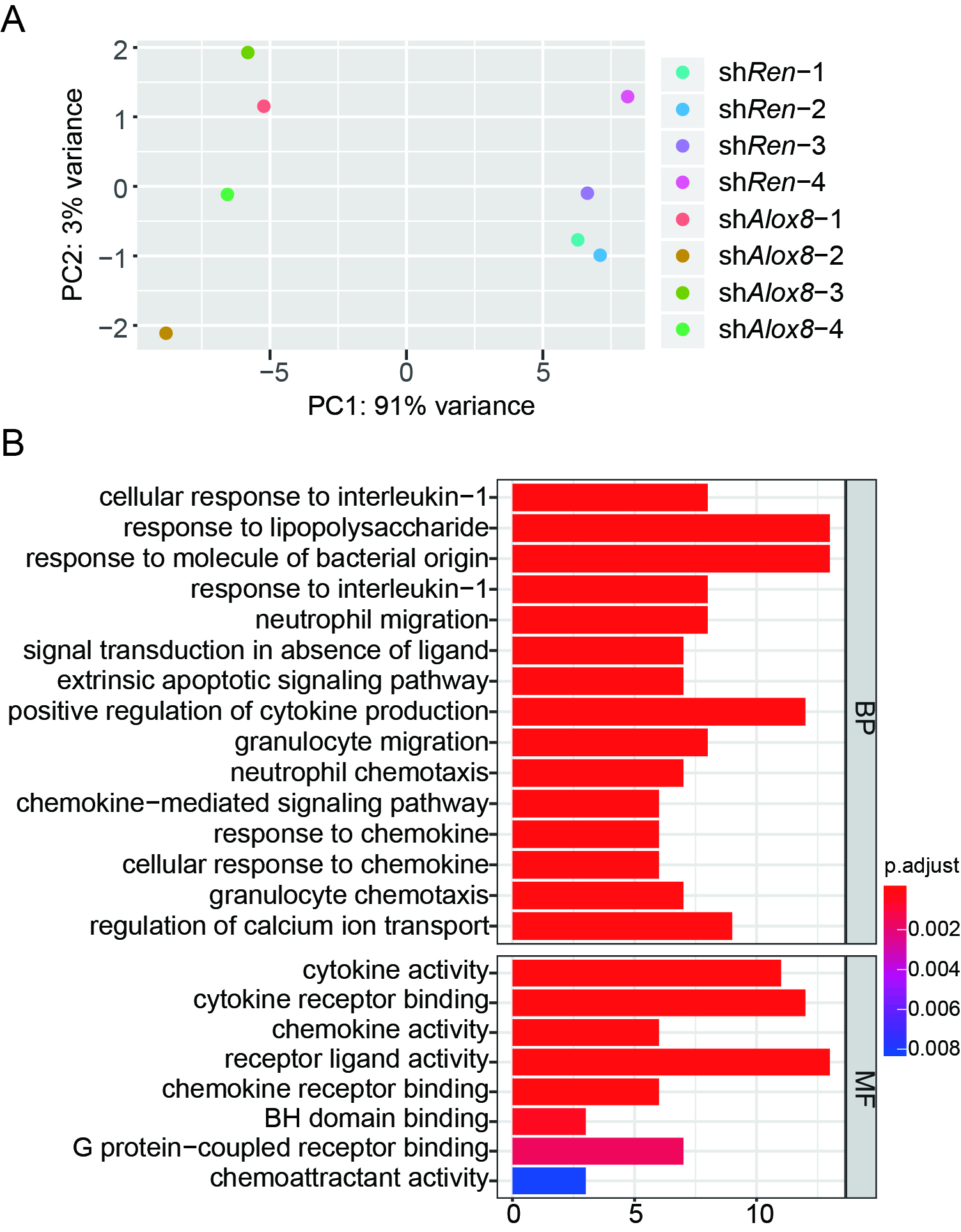

Supplement: Supplementary file 2 — Supplemental figure 2 [file 41389_2023_451_MOESM2_ESM.jpg]

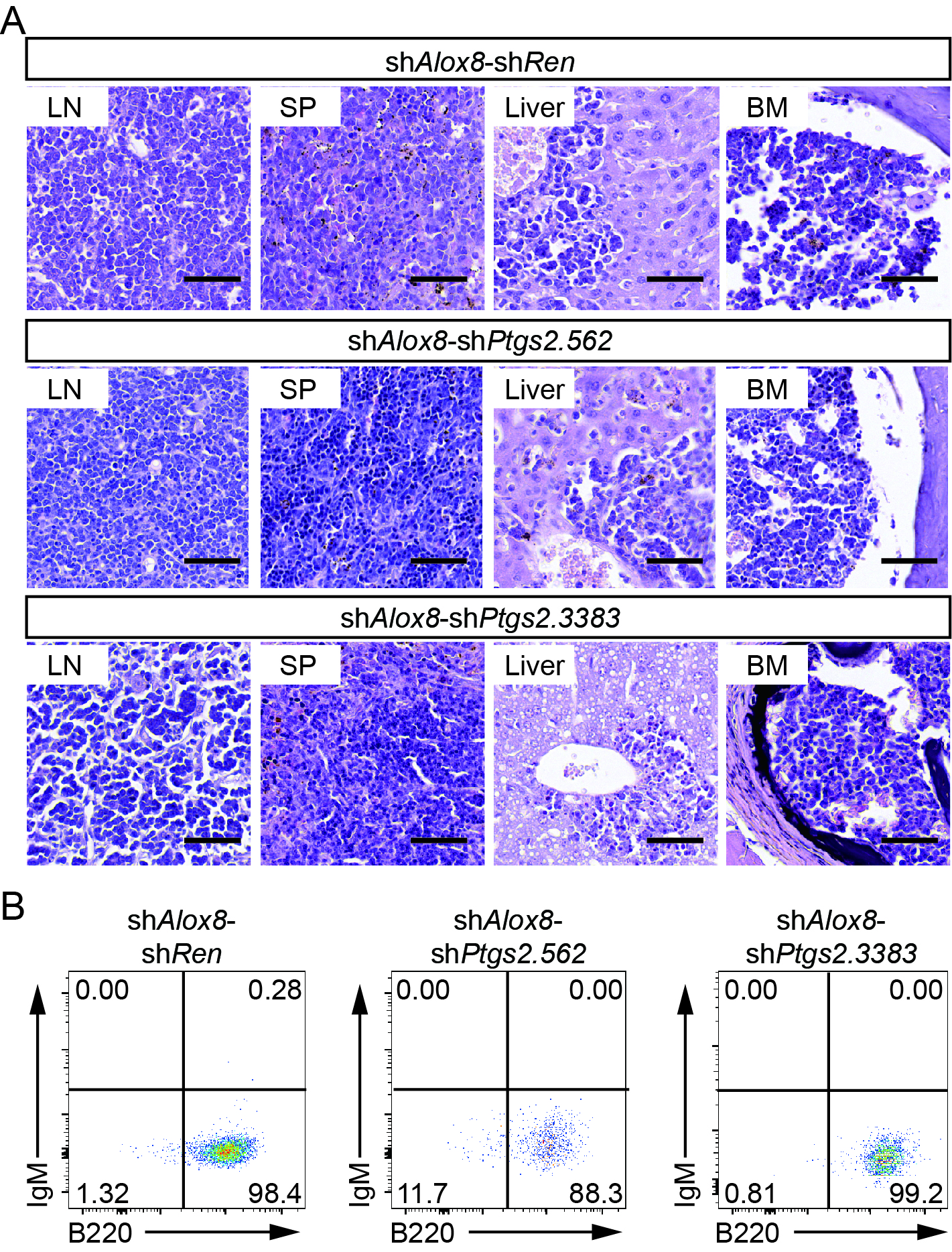

Supplement: Supplementary file 3 — Supplemental figure 3 [file 41389_2023_451_MOESM3_ESM.jpg]
