# Supplementary material for: COX-2/PGE2 upregulation contributes to the chromosome 17p-deleted lymphoma
Source: Oncogenesis. 2023 Feb 7;12(1):5. doi: 10.1038/s41389-023-00451-9 (PMC9905509; doi:10.1038/s41389-023-00451-9)
Supplement: Supplementary file 4 — Supplementary figure legends [file 41389_2023_451_MOESM4_ESM.docx]

**Supplementary Figure 1.** **Generate *Alox15b* loss-of-function mutated Ba/F3 cells by CRISPR/Cas9.** (A) AA levels in sh*Alox15b* vs sh*Ren* Ba/F3 cell culture medium, measured by ELISA, respectively. Error bar represents. “Mean with SEM”, **p<0.01 (unpaired two-tailed *t-*test). (B) AA levels in sg*Alox15b* vs sg*Scr* Ba/F3 cell culture medium, measured by ELISA, respectively. “Mean with SEM”, *p<0.05 (unpaired two-tailed *t-*test). (C) T7 endonuclease I assay performed after on genomic DNA from Ba/F3 cells infected with sg*Alox15b* and Cas9. Arrowheads show cleaved fragments. UT, untreated cells (negative control). (D) Sequence alignment of the wildtype allele and mutant alleles from each Ba/F3 cell line with sg*Alox15b*, detected by Sanger sequencing.

**Supplementary Figure 2.** **RNA-sequence analysis of pre-B cells infected by sh*Alox15b* or sh*Ren*.** (A) PCA analysis of the transcriptome of sh*Ren* and sh*Alox15b* pre-B cells. (B) GO enrichment analysis of gene expressions in sh*Alox15b* pre-B cells, compared to that in sh*Ren* control cells.

**Supplementary Figure 3.** **Pathology of sh*Alox15b*_sh*Ptgs2* or sh*Alox15b*_sh*Ren* lymphomas.** (A) Hematoxylin and eosin stains of LN, SP, liver and BM of moribund, lymphoma-bearing recipient mice transplanted with sh*Alox15b*_sh*Ptgs2* or sh*Alox15b*_sh*Ren* and *Myc* infected pre-B cells. Bar, 50μm. LN, lymph node; SP, spleen; BM, bone marrow. (B) Immunophenotypes of B220+IgM- lymphoma cells harvested from enlarged lymph nodes of above recipient mice (A).
