# Supplementary material for: COX-2/PGE2 upregulation contributes to the chromosome 17p-deleted lymphoma
Source: Oncogenesis. 2023 Feb 7;12(1):5. doi: 10.1038/s41389-023-00451-9 (PMC9905509; doi:10.1038/s41389-023-00451-9)
Supplement: Supplementary file 5 — Supplemental tables [file 41389_2023_451_MOESM5_ESM.docx]

Supplementary Table 1. abbreviations of AA-derived eicosanoids

| **Abbreviation** | **Full Name** |
| --- | --- |
| (±)10-HDoHE | 10-hydroxy docosahexaenoic acid |
| 11,12-EET | 11,12-epoxy-eicosatrienoic acid |
| 11-HETE | 11-hydroxy-eicosatetraenoic acid |
| 12,13-EpOME | 12,13-epoxyoctadecenoic acid |
| 12-HEPE | 12-Hydroxy-eicosapentaenoic acid |
| 12-HETE | 12-hydroxy-eicosatetraenoic acid |
| (±)13-HDoHE | 13-hydroxy docosahexaenoic acid |
| 13-HODE | 13-hydroxyoctadecadienoic acid |
| 15d-12,14-PGD2 | 15­deoxy­Δ12,14­Prostaglandin D2 |
| 15-HEPE | 15-Hydroxy-eicosapentaenoic acid |
| 15-HETE | 15-hydroxy-eicosatetraenoic acid |
| 15(S)-HpETE | 15S­hydroperoxy­5Z,8Z,11Z,13E­eicosatetraenoic acid |
| 19,20-EDP | 19,20-epoxy docosapentaenoic acid |
| (±)20-HDoHE | 20-hydroxy docosahexaenoic acid |
| (±)4-HDoHE | 4-hydroxy docosahexaenoic acid |
| 5-HETE | 5-hydroxy-eicosatetraenoic acid |
| (±)8-HDoHE | 8-hydroxy docosahexaenoic acid |
| 8-HETE | 8-hydroxy-eicosatetraenoic acid |
| 9,10-EpOME | 9,10-epoxyoctadecenoic acid |
| 9-HODE | 9-hydroxyoctadecadienoic acid |
| AA | Arachidonic Acid |
| DHA | Docosahexaenoic Acid |
| DPA | Docosapentaenoic Acid |
| EPA | Eicosapentaenoic Acid |
| PGA2 | Prostaglandin A2 |
| PGD2 | Prostaglandin D2 |
| PGE2 | Prostaglandin E2 |
| PGF2α | Prostaglandin F2alpha |
| TXB2 | Thromboxane B2 |

Supplementary Table 2. Sequences of qRT-PCR primers.

| Primer name | Primer sequence |
| --- | --- |
| Cox1-F | GGTAGTTGTCGAGGCCAAAG |
| Cox1-R | GTCCTGCTCGCAGATCCT |
| Cox2-F | GGCGCAGTTTATGTTGTCTGT |
| Cox2-R | CAAGACAGATCATAAGCGAGGA |
| Hprt-F | TGACTGATCATTACAGTAGCTCTTC |
| Hprt-R | CATTGTGGCCCTCTGTGT |

Supplementary Table 3. 97nt sequences of shRNAs.

| Name | Sequence |
| --- | --- |
| shCox2.562 | TGCTGTTGACAGTGAGCGCAAGGAGCTTCCTGATTCAAAATAGTGAAGCCACAGATGTATTTTGAATCAGGAAGCTCTTATGCCTACTGCCTCGGA |
| shCox2.3383 | TGCTGTTGACAGTGAGCGCTAGGGGAGTTCCTGACAAGAATAGTGAAGCCACAGATGTATTTCTTGTCAGGAACTCCCTATTGCCTACTGCCTCGGA |
